# The effectiveness of narrative enhancement and cognitive therapy: a randomized controlled study of a self-stigma intervention

**DOI:** 10.1007/s00127-017-1385-x

**Published:** 2017-04-19

**Authors:** Lars Hansson, Annika Lexén, Joacim Holmén

**Affiliations:** 1 Department of Health Sciences, Lund University, PO Box 157, 22100 Lund, Sweden; 2 Department of Psychiatry, Sahlgrenska University Hospital, 413 45 Gothenburg, Sweden

**Keywords:** Self-stigma, NECT, Anti-stigma intervention, Severe mental illness, Recovery

## Abstract

**Purpose:**

Stigma has been proposed to be one of the most serious obstacles to successful treatment, rehabilitation and inclusion in society of people with severe mental illness. An aspect of stigma which has been increasingly discussed is self-stigma, which refers to the internalization of negative stereotypes among people with severe mental illness. The aim of the present study was to investigate the effectiveness of a group-based anti self-stigma intervention, narrative enhancement and cognitive therapy (NECT) as an add-on to treatment as usual, with regard to changes in self-stigma, self-esteem, and subjective quality of life.

**Method:**

After screening for eligibility 106 participants were included in a randomized controlled trial using a wait-list control group, of which 87 completed the study. Assessments were made at baseline, at termination of the intervention, and at a 6-month follow-up (intervention group only).

**Results:**

The results showed that NECT was effective in reducing self-stigma and improving self-esteem compared to treatment as usual only. No differences were shown regarding subjective quality of life. Changes shown in the intervention group at termination of intervention were stable at the 6-month follow-up. A regression analysis showed that there was a positive relationship between exposure to the intervention and reduction of self-stigma.

**Conclusions:**

The conclusion of the present study is that, using a sample size with adequate power, NECT seems to be an effective intervention with regard to diminishing self-stigma and improving self-esteem, and that these improvements were stable at a 6-month follow-up. There was a distinct relationship between number of sessions attended and improvements in self-stigma and self-esteem controlling for confounding factors. This puts attention to the importance of creating a group climate which facilitate and encourage participation through the various phases of the intervention.

## Introduction

Although recent anti-stigma programs have shown small to moderate effects [1, 2], it is still not clear whether these have a lasting impact [3]. Public stigma against people with mental illness is, therefore, still highly prevalent and the main conclusion is that it has not diminished during the last decades [4]. Stigma has been proposed to be perhaps the most serious obstacle to a successful treatment, rehabilitation, and inclusion of people with mental illnesses in society. Unemployment, income loss [5], not seeking care or delayed care [6] a diminished social network [7, 8], impaired self-esteem [9], isolation and loneliness have been associated with stigma and discrimination. Stigma also affects disease progression and recovery [10].

The generally expanding scientific literature on mental illness stigma has so far no correspondence in studies on discrimination and self-stigma, where there still is a dearth of studies. Discrimination deals with people’s behaviour as captured by observational studies, by studies of structural discrimination, for example related to the judicial system, or by studies focusing on the experiences of people with mental illness. The INDIGO study (International Study of Discrimination and Stigma Outcomes) performed a quantitative cross-sectional study of people with schizophrenia covering 27 countries which showed that perceived discrimination was common in a number of areas and most prevalent in areas of making or keeping friends, family members, and in finding and keeping a job [11]. A majority of the participants also reported anticipated discrimination in applying for work/education and making close relationships. Almost 75% of the participants concealed their diagnosis to their social network. A further study from the INDIGO/ASPEN groups focusing discrimination in people with depression including 1082 people also showed that perceived discrimination was common, reporting that 79% had perceived discrimination in at least one area. This study also showed that anticipated discrimination was not always associated with actually perceived discrimination, almost half of the people reporting anticipated discrimination regarding finding and keeping a job or in their intimate relationships actually did not report any perceived discrimination in these areas [12]. The GAMIAN study (Global Alliance of Mental Illness Advocacy Networks study) made a similar cross-country survey including people with schizophrenia and bipolar disorder [13, 14]. This study focused more on perceived anticipated stigma and self-stigma. They reported, for both conditions, that a majority had moderate or high perceived discrimination, that almost half of people with schizophrenia reported moderate or high levels of self-stigma, and that the equivalent figure for people with bipolar disorders was around one-fifth of the participants.

The internalisation of negative stereotypes about mental illness occurs early in life and may lead to the development of self-stigma for people afflicted by mental illness later on in life. Self-stigma (or internalized or felt stigma) exists on the individual level and indicates that the individual endorses stereotypes of mental illness, finds these stereotypes relevant and anticipates social rejection [15]. Self-stigma is highly prevalent in people with longstanding and severe mental illness; a review of studies investigating the prevalence of elevated levels of self-stigma [16] showed prevalence rates in the range of 27–49%. Self-stigma may also be a response to actual experiences of public stigma and discriminatory behaviour, which could result in consequences in a number of psychosocial life aspects: refraining from applying for work, avoiding contact with mental health care and social contacts [17, 18]. In a review by Gulliver and colleagues [19] public stigma and self-stigma was found to be the two most important perceived barriers for people with mental health issues to actually seek help. The review by Livingstone and Boyd [18] draws attention to the fact that, while there is a rather vast scientific literature on self-stigma and its correlates, there is a lack of studies with a longitudinal design which may have increased the clinical value of this literature and increased opportunities to develop and engage in research focusing interventions to cope with self-stigma.

Despite the substantial evidence for the negative effects of self-stigma, the development of interventions to decrease self-stigma is a relatively new area of research. A recent review of self-stigma reduction interventions included 14 studies, of which eight reported significant improvements in self-stigma outcomes [20]. Six self-stigma reduction strategies were identified of which psychoeducation was the most frequently tested type of intervention. Two prominent approaches for self-stigma reduction emerged: interventions that attempt to alter the stigmatizing beliefs and attitudes of the individual; and interventions that enhance skills for coping with self-stigma through improvements in self-esteem, empowerment, and help-seeking behaviour. A further review by Yanos et al. showed similar results [21]. They conclude that several interventions show a positive impact and that several interventions are in the process of rigorous studies, including larger RCT’s. However, outcome and implementation research is clearly in early stages; further evaluation is needed to understand the potential of many programs.

One of the interventions included in the Mittal review was the narrative enhancement and cognitive therapy intervention (NECT) [22]. It is a rather comprehensive intervention which is attractive since it includes both the major approaches that emerged from the Mittal et al. review, and in addition a module aiming at changing the person´s self-concept. Although NECT seems to be a comparatively promising intervention in terms of its evidence-base there are so far a few investigations focusing outcome of NECT. Qualitative analyses of interviews conducted with 18 NECT completers showed that they perceived NECT as helpful. Six domains of improvement were revealed which participants attributed to participating in the intervention: experiential learning, positive change in experience of self, acquiring cognitive skills, enhanced hope, and coping and emotional change [23]. A small RCT has also been performed [24], 39 persons with SMI were randomized to NECT or to treatment as usual and were assessed at baseline, post treatment, and at a 3-month follow-up. Fifteen of the 21 individuals assigned to NECT were classified as “exposed” to treatment and, compared to unexposed participants, improved more in two aspects of self-stigma as well as insight; however, the small sample size and significant dropout rates restricted the ability to detect an effect. A quasi-experimental study involving 109 persons with severe mental illness revealed that the NECT group showed significant reductions in self-stigma and improvements in self-esteem, quality of life and hope between pre- and post-treatment assessments, compared with the control group [25]. A non-controlled pilot study, performed as part of the implementation of NECT in Sweden, showed a decrease of self-stigma and improvements in self-esteem and quality of life [26].

So far no randomized controlled trial using a sample with sufficient power to investigate the effectiveness of NECT has been performed. Based on the positive results of the Swedish pilot study the aim of the present study was to investigate the effectiveness of NECT in reducing self-stigma and improving self-esteem and subjective quality of life.

## Methods and participants

### Design of the trial

The intervention was given as an add-on to treatment as usual. The study used a randomized controlled design, using waiting list participants as control group. Patients in the setting where the intervention was performed were before the study subject to a screening procedure performed by staff at the participating units as part of the clinical work and used as a tool for the selection of patients to the NECT groups. The screening was performed during a 6-week period in spring 2015 by using a scale assessing levels of self-stigma developed by Corrigan et al. (SSMIS-SF) [27]. Patients scoring above a defined cut-off score was informed about the study and invited to participate. Those giving informed consent was after baseline assessments randomized to the intervention group or the control group consisting of a 6-month waiting list group. Randomization was performed by an independent researcher using procedures from http://www.randomizer.org/. Further inclusion criterion was an ability to read and speak Swedish since participant manuals are only available in Swedish.

Based on the results from the pilot study investigating the first NECT groups in Gothenburg [26] a power calculation was performed. Using assessments from this study regarding self-stigma (primary outcome measure) an effect size of 0.6 with a power of 80% and level of significance set at *p* < 0.05 would require a total sample of 96 patients. Accounting for a drop-out of around 15%, the aim thus was to include 115 participants in the study. This was also the maximum number of participants the services could include in the NECT groups due to limitations in the number of trained group leaders available.

Participants were assessed at baseline, post intervention and at a 6-month follow-up (intervention group only) regarding self-stigma, quality of life, and self-esteem. Sociodemographic and clinical background characteristics were obtained at baseline. A registration of exposure to the intervention was performed (number of sessions participants were attending the group).

The research questions were:

1. Is NECT effective in decreasing self-stigma as compared to treatment as usual only (primary outcome measure)?
2. Is NECT effective in increasing self-esteem, and quality of life as compared to treatment as usual only (secondary outcome measures)?
3. Is exposure to the intervention (number of sessions attended) related to outcome?

### Participants

All in all 324 patients were screened using the SSMIS-SF, of which 236 were eligible for the intervention since they scored above the cut-off point in the screening questionnaire. Due to the limitations in the number of NECT groups possible to run by the clinical services not all of these were asked for participation and informed about the study. Inclusion was stopped when 106 patients had given informed consent and these were randomized into intervention or waiting list control group, 53 participants in each group. There were no significant difference regarding self-stigma between those 236 eligible for inclusion and those giving informed consent to participation (*t* = 0.55; *p* = 0.58).

**Fig. 1:**
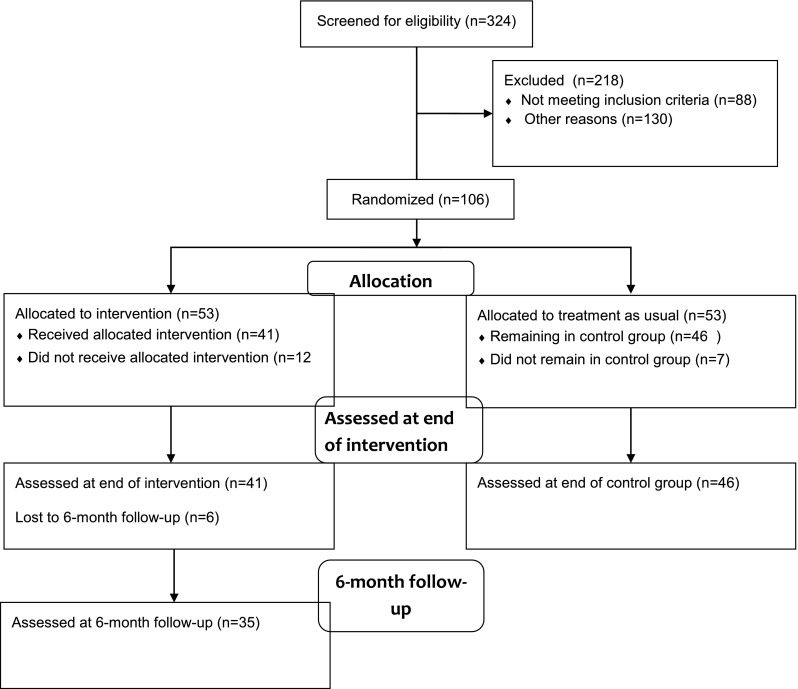
CONSORT flow diagram

Nineteen participants dropped out of the study between pre- and post-treatment assessments of the intervention group, 12 from the intervention group and seven from the control group, leaving 87 (82%) participants remaining in the study, 41 in the intervention group and 46 in the control group. Participants withdrawing from the study did not in any of the two groups differ from those remaining with regard to background characteristics or baseline assessments of outcome measures. For information about the flow of patients in the study se Fig. 1.

Fourteen of the 19 participants withdrawing during the intervention phase accepted to be interviewed regarding reasons for withdrawal. Eight participants never participated in any of the group sessions, one participant dropped out after a few sessions, two participants moved to another city, and three participants felt that the intervention worsened their mental health status and, therefore, dropped out. A further six participants in the intervention group withdraw from the 6-month follow-up study, thus 35 participants, 85% of those remaining at termination of the intervention were assessed at this point.

Background characteristics of the included sample are shown in Table 1. There were no baseline differences between the groups regarding social and clinical characteristics or regarding baseline assessments in any of the outcome measures.

**Table 1:** Background characteristics of participants (*N* = 106)

Variable	Intervention group		Control group	
	*N* (53)	%	*N* (53)	%
Sex				
Male	25	47.2	27	50.9
Female	28	52.8	26	49.1
Age (m, sd)	45.1	11.5	45.3	10.9
Living situation				
Alone	35	70	34	65.4
Partner	5	10	13	25
Parents	3	6	4	7.7
Other	7	14	1	1.9
Education				
Primary school	6	11.3	9	17
High school	26	49.1	20	37.7
University	21	39.6	24	45.3
Working situation				
Working	14	26.9	10	18.9
Unemployed	14	26.9	14	26.4
In education	23	44.2	27	50.9
Pension	1	1.9	2	3.8
Contacts with mental health care				
No. of years (m, sd)	18.7	10.2	17.4	13.6
No. of inpatient admissions (m, sd)	4.0	4.7	3.2	3.5
Involuntary admissions (yes)	25	47.2	30	56.6
Diagnosis (*N* = 76)^a^				
Psychosis	24	64.9	28	71.8
Depression, anxiety	6	16.2	6	15.4
Other	7	18.9	5	12.8

^a^Self-reported diagnosis available for 76 participants (71.6%)

### Intervention: narrative enhancement and cognitive therapy

NECT is a structured, group-based intervention that combines (a) psychoeducation to help replace stigmatizing views about mental illness and recovery with empirical findings, (b) cognitive restructuring geared toward teaching skills to challenge negative beliefs about the self, and (c) narrative therapy focused on enhancing one’s ability to narrate one’s life story [22]. NECT has a focus on facilitating the transformation of personal narratives based on phenomenological observations that having a severe mental illness often involves a diminishment in a person’s ability to narrate his or her own life’s evolving story. This may concern temporal organization or having difficulties in differentiating themselves from their disorder. Narrative transformation is in this context considered to be an essential tool for the process of identity transformation. NECT attempts to help individuals transform their narratives by engaging participants in a series of story-telling exercises where they are encouraged to write or dictate stories about themselves, and then receive feedback from the facilitator and group members on alternative perspectives regarding the themes that their stories contain.

The format of NECT is a 20-session manualized intervention including 4 sections: (1) Introduction (starting the process of assessing where the participant is with regard to experience of self and self-stigma), (2) Psychoeducation (providing participants with information about the inaccuracy of stigmatizing views about SMI), (3) cognitive restructuring (teaching the basic principles of cognitive restructuring and encouraging participants to apply these techniques to self-stigmatizing cognitions), and (4) narrative enhancement (where participants are encouraged to write and share stories within the group, while focusing on trying to bring together previously fragmented and isolated aspects of the self) [23]. It is recommended that the group be conducted by two facilitators. The ideal group size is 6–8 participants, not including facilitators, and each group meeting should last for 1 h. There is a manual acting as a guide for the facilitator, as well as a manual to be used by the group participants. These manuals have been translated into Swedish. There is also a fidelity scale for NECT to be used by an external observer.

### Setting and implementation

The study was performed at the psychosis mental health services in Gothenburg. These services cover Gothenburg city and the surrounding local municipalities. The services contain four inpatient units and seven outpatient units; altogether in contact with 2600 patients. The services offer as treatment as usual a comprehensive treatment and collaboration including medical, psychological and psychosocial interventions according to evidence-based care principles. Professor Philip Yanos, one of the developers of NECT, was invited and held a 1 day training session in Gothenburg including almost 30 clinicians. These constituted the core group who later performed the intervention.

### Outcomes

Self-stigma was assessed by the Self-Stigma of Mental Illness Scale-Short Form (SSMIS-SF) [27]. The measure is a self-report questionnaire divided into four subscales representing awareness (e.g., ‘‘I think the public believes most persons with mental illness are dangerous.’’), agreement (e.g. ‘‘I think most persons with mental illness are dangerous.’’), application (e.g. ‘‘Because I have a mental illness, I am dangerous.’’), and harm to self-esteem (e.g. ‘‘I currently respect myself less because I am dangerous.’’). Each subscale has 5 items and participants respond to items using a nine point agreement scale (1 = totally disagree; 9 = strongly agree). Scale scores are determined by summing the five items for each subscale, yielding a range of scores between 5 and 45 for each of the four subscales. A summary score may also be obtained. Results from a psychometric study indicate adequate internal consistencies for each subscale. Validity was supported by inverse and significant relationships, to self-esteem, self-efficacy, empowerment, and hope [27]. The SISMIS-SF has been translated into Swedish and shown good psychometric properties [28]. Internal consistency in the present study ranged between 0.86 and 0.90.

Self-esteem was measured using the Rosenberg self-esteem scale [29]. The scale is a self-report questionnaire and consists of 10 items which respondents answer using a 4-point scale. Item responses are summed into a total score. The scale has shown a satisfactory validity, internal consistency and good test retest reliability [30, 31]. Internal consistency ranged in the present study between 0.87 and 0.89.

Manchester Short Assessment of Quality of Life (MANSA) was used to assess quality of life. It contains 16 questions, four of them assessing objective quality of life and 12 assessing satisfaction with life as a whole, job, financial situation, friendships, leisure activities, accommodation, personal safety, sex life, people the person live with, family and health. Satisfaction is rated on a 7-point scale ranging from 1 = could not be worse to 7 = could not be better, and an overall score of subjective quality of life may be calculated. The Swedish version of MANSA has been tested for reliability and validity with satisfactory results [32]. In the present study internal consistency ranged between 0.82 and 0.88.

Clinical and sociodemographic background characteristics were also obtained at baseline including a self-reported diagnosis.

### Statistics

Repeated measures of ANOVA were used for analyses of differences between intervention group and control group over time. Paired sample *t* tests were used to compare within intervention group changes between baseline and the 6-month follow-up. Chi-square tests and independent sample *t* tests were used to check for differences between dropouts and those remaining in the study. Spearmen rank correlations and stepwise regression analyses were performed to investigate the impact of exposure to the intervention. Effect sizes (ES) were calculated using Cohen’s *D*. Post-hoc power analyses revealed a power of 0.81 to detect the actual effect size in the primary self-stigma outcome measure. Cronbach’s alpha was used to calculate reliability in terms of internal consistency.

### Ethics

All instruments used in the present study have been tested and used in the target population with no reports of problems related to ethical issues. However, some of the instruments contain questions which might be viewed by the participants as focusing personal and private issues, therefore, the research assistant in charge of the data collection was available to take care of problems related to this. Participation was based on informed consent and the study was approved by the Regional Research Ethical Review Board in Lund, Sweden (DNR 2015/414), and in all aspects been performed in accordance with the ethical standards in the 1964 Declaration of Helsinki and its later amendments.

## Results

Analyses of the pre-post intervention changes showed that the intervention group improved significantly more than the control group in the primary outcome measure self-stigma (ES = 0.5). There were also significant differences between the groups in favour of the intervention group in three of the subscales of SSMIS-SF, Awareness, Agreement and Application, but not in the subscale measuring Harm to the self, Table 2. There were also a significant improvement in self-esteem in the intervention group as compared to the control group (ES = 0.5) but no difference with regard to quality of life, although there was a tendency in favour of the intervention group (*p* = 0.09), Table 3.

**Table 2:** Changes in self-stigma pre-post intervention in the NECT group and control group (*N* = 87)

Subscale SSMIS	Group	Pre m, sd	Post m, sd	*p* value^a^	Effect size	95% CI
Awareness	Intervention	29.26 ± 6.34	24.93 ± 8.46	0.049		
	Control	29.48 ± 6.54	28.90 ± 7.78			
Agreement	Intervention	18.83 ± 5.71	14.60 ± 7.34	0.028		
	Control	18.80 ± 8.74	18.26 ± 7.67			
Application	Intervention	16.75 ± 7.10	13.95 ± 7.74	0.042		
	Control	15.98 ± 7.16	16.33 ± 8.40			
Harm	Intervention	16.66 ± 8.43	13.53 ± 8.71	ns		
	Control	17.69 ± 9.20	16.27 ± 9.22			
Overall score	Intervention	81.49 ± 20.13	67.00 ± 24.60	0.013	0.5	0.04–0.89
	Control	82.07 ± 23.71	79.88 ± 26.48			

^a^Repeated measures ANOVA

**Table 3:** Changes in self-esteem and subjective quality of life pre-post intervention in the NECT group and control group (*N* = 87)

Measure	Group	Pre	Post	*p* value^a^	Effect size	95% CI
Self-esteem	Intervention	24.77 ± 6.63	27.10 ± 5.48	0.008	0.5	0.01–0.84
	Control	25.20 ± 6.41	24.75 ± 5.74			
Quality of life	Intervention	50.62 ± 10.58	54.55 ± 12.50	ns		
	Control	51.34 ± 11.10	52.11 ± 11.24			

^a^Repeated measures ANOVA

A 6 month after termination of the intervention follow-up was also performed. Comparisons between baseline and follow-up showed significant improvements in self-stigma and self-esteem but no changes in quality of life, indicating that improvements at termination of intervention were stable over the follow-up period, Table 4. There were no significant changes between termination of intervention and follow-up in any of the measures.

**Table 4:** Changes in self-stigma, self-esteem and quality of life between baseline and 6-month follow-up in the intervention group (*N* = 35)

Scale	Baseline m, sd	Follow-up m, sd	*p* value*	Effect size	95% CI
Self stigma	79.71 ± 17.27	68.40 ± 21.76	0.001	0.58	0.09–1.05
Self-esteem	25.57 ± 6.52	28.30 ± 5.82	0.008	0.44	0.04–0.91
Quality of life	51.43 ± 9.80	52.76 ± 12.90	NS		

* Paired sample *t* test

Mean number of sessions attended for those remaining in the intervention group at termination (*N* = 41) was 14 (range 4–20). To explore whether exposure to the intervention in terms of number of sessions attended was related to outcome, correlations between number of sessions and changes in self-stigma were performed. The covariation between exposure and changes in self-stigma was substantial (*r* = 0.49; *p* = 0.001), indicating an explained variance of changes in self-stigma of around 25%. To further explore this we performed a stepwise regression analysis using ratings of self-stigma at termination of intervention as dependent variable and controlling for self-stigma at baseline and the clinical background variables number of years in contact with mental health services, number of inpatient episodes and history of involuntary care or not. This analysis showed that the two variables entering the model were self-stigma at baseline (*t* = 3.07; *p* = 0.004), explaining 14.1% of the variance in self-stigma at termination, and exposure (*t* = 4.20; *p* = 0.001), explaining a further 30.6% of the variance.

## Discussion

We have earlier performed an open trial of NECT in Sweden, not including any control group [23]. A conclusion from this study, on background of the encouraging results, was that the next step would be to set up an RCT to more adequately evaluate the effectiveness of NECT. In the present study, we included 106 participants, based on a power analysis relying on the results from the earlier open trial. Of these 87 remained in the study at termination of the intervention period, giving a reasonable attrition rate of 18%. Interviews with the drop-outs showed that rather few referred to negative aspects of the intervention as reason for withdrawal. The vast majority referred to various practical circumstances as reason for not attending the group sessions. A drop-out analysis also showed that the drop-outs did not differ from those remaining with regard to background characteristics or baseline assessments of outcome measures. Furthermore, we did not detect any baseline differences between the intervention and control group regarding background characteristics or outcome measures. We may, therefore, assume that completers constitute a group fairly representative for the included sample.

This is the so far largest RCT performed of NECT. The main results were that NECT was effective in reducing self-stigma and improving self-esteem in comparisons with the control group, in both cases with lower end moderate effect-sizes. The only exception regarding self-stigma was the subscale “harm to self” were no difference was found. The main reason for this may be that this subscale, according to Corrigan’s model [27], rates the most severe and invasive consequences of self-stigma, and thus probably is the hardest to change since it indicates the most severe damage to a person’s self-concept. Furthermore, these effects seem stable over a 6-month follow-up period after the termination of intervention. These results are in contrast with the only former RCT performed, which showed no superiority of NECT versus treatment as usual only [24]. However, this study was underpowered which may be part of the explanation for this difference. The reason for no difference regarding quality of life may have a number of reasons including the intervention not specifically targeting subjective quality of life or the way we assessed quality of life, but may also more generally reflect that quality of life in many community mental health intervention studies have not shown to be a sensitive outcome measure [33].

An interesting question is if any dose–response relationships exist. Findings from our earlier pilot study [26] indicated this, as well as did the RCT study by Yanos et al. [24]. These findings were verified by the present study, which showed a distinct relationship between number of sessions attended and improvements in self-stigma and self-esteem. A further regression analysis revealed that this was not due to any potential confounders such as various aspects of severity of illness. This finding puts attention to the importance of creating a group climate which facilitate and encourage participation through the various phases of the intervention. This is also corroborated by the results from a qualitative study of NECT [23] where completers of NECT assigned helpfulness to various domains of the intervention.

A limitation of the present study is that not all patients fulfilling inclusion criteria could be included in the study due to the limitations in the number of NECT groups able to manage by the service. We have no information of whether there is a possible bias between those informed and giving consent and those not asked for participation. The information given to clinical staff was that inclusion should be made on a “first come first serve basis”. Comparisons between all patients fulfilling inclusion criteria and those included did, however, not show any significant differences with regard to the level of self-stigma. We, therefore, tentatively assume that study participants are representative for those fulfilling inclusion criteria. A further limitation is that we do not have consistent information about the number of patients who were asked for participation but refused; they are included in the number not asked for participation.

The conclusion of the present study is that, using a sample size with adequate power, NECT seems to be an effective intervention with regard to diminishing self-stigma and improving self-esteem, but did not show any improvement in subjective quality of life. The sample used in the study had a rather long duration of illness. Analysis of the correlations between outcome regarding self-stigma and duration of illness showed that they were low and non-significant. We, therefore, aim to set up an RCT including only first episode psychosis patients to test the hypothesis that NECT is effective in this group. If this hypothesis will be verified NECT may be used in first episode psychosis services to prevent or diminish the long standing negative and serious consequences of self-stigma.
